# Association Between Diverse Cell Death Patterns Related Gene Signature and Prognosis, Drug Sensitivity, and Immune Microenvironment in Glioblastoma

**DOI:** 10.1007/s12031-023-02181-4

**Published:** 2024-01-12

**Authors:** Jian Li, Zhaoming Song, Zhouqing Chen, Jingyu Gu, Yifan Cai, Li Zhang, Zhong Wang

**Affiliations:** 1 Department of Neurosurgery, Zhangjiagang Hospital affiliated to Soochow University/ The First Peoples’ Hospital of Zhangjiagang City, Suzhou, China; 2 Department of Neurosurgery and Brain and Nerve Research Laboratory, The First Affiliated Hospital of Soochow University, Suzhou, 215006 China

**Keywords:** Glioblastoma, Programmed cell death, Prognosis, Drug sensitivity, Tumor microenvironment

## Abstract

Glioblastoma (GBM) is the most invasive type of glioma and is difficult to treat. Diverse programmed cell death (PCD) patterns have a significant association with tumor initiation and progression. A novel prognostic model based on PCD genes may serve as an effective tool to predict the prognosis of GBM. The study incorporated 11 PCD patterns, namely apoptosis, necroptosis, pyroptosis, ferroptosis, cuproptosis, entotic cell death, netotic cell death, parthanatos, lysosome-dependent cell death, autophagy-dependent cell death, alkaliptosis, and oxeiptosis, to develop the model. To construct and validate the model, both bulk and single-cell transcriptome data, along with corresponding clinical data from GBM cases, were obtained from the TCGA-GBM, REMBRANDT, CGGA, and GSE162631 datasets. A cell death-related signature containing 14 genes was constructed with the TCGA-GBM cohort and validated in the REMBRANDT and CGGA datasets. GBM patients with a higher cell death index (CDI) were significantly associated with poorer survival outcomes. Two separate clusters associated with clinical outcomes emerged from unsupervised analysis. A multivariate Cox regression analysis was conducted to examine the association of CDI with clinical characteristics, and a prognostic nomogram was developed. Drug sensitivity analysis revealed high-CDI GBM patients might be resistant to carmustine while sensitive to 5-fluorouracil. Less abundance of natural killer cells was found in GBM cases with high CDI and bulk transcriptome data. A cell death-related prognostic model that could predict the prognosis of GBM patients with good performance was established, which could discriminate between the prognosis and drug sensitivity of GBM.

**Supplementary Information:**

The online version contains supplementary material available at 10.1007/s12031-023-02181-4.

## Introduction

Glioblastoma (GBM) is the most common form of malignant primary brain cancer, affecting approximately two to five people per 100,000 cases annually in the USA and Europe (Bikfalvi et al. 2023). Despite the administration of surgery and the Stupp protocol combining radiotherapy with concomitant chemotherapy with temozolomide (TMZ) in GBM, the prognosis for GBM remains poor, as patients typically experience a median survival period of approximately 15 months after treatment (Fabbro-Peray et al. 2019). Blood–brain barrier, immune escape, and tumor heterogeneity may lead to therapy resistance for GBM, while the potential mechanisms of GBM cell treatment escaping and invasion are incompletely understood (Gonzalez Castro et al. 2021). A good prognostic model for GBM may help in the development of individualized and effective treatment strategies. Additionally, identifying robust underlying treatment targets to design optimized drugs is also urgently needed.

Cell demise can be classified according to morphological characteristics, the cellular environment, and the stimulus that triggers the procedure, primarily encompassing programmed cell death (PCD) and unintentional cell death (ACD). ACD is an unregulated process whereby cells succumb to death in response to stimuli resulting from accidental injuries. Cell death occurs when the cellular ability to adapt or adjust is surpassed by these injury stimuli, resulting in their ultimate demise (Galluzzi et al. 2018). PCD refers to a self-regulated and organized process by which cells undergo demise under the control of specific genes. PCD’s main goal is to uphold the stability of the internal cellular environment (Galluzzi et al. 2018). PCD encompasses various forms of cell death, such as apoptosis, pyroptosis, ferroptosis, autophagy, necroptosis, cuproptosis, parthanatos, entotic cell death, netotic cell death, lysosome-dependent cell death, alkaliptosis, and oxeiptosis (Tang et al. 2019). Various pathways and their crosstalk during PCD can significantly influence tumor progression and response to anti-cancer treatment. Apoptosis, which has been extensively researched, has been considered the sole form of regulated cell death for many years. The interplay among different proteins of the BCL-2 family, comprising both proapoptotic and antiapoptotic members, governs the crucial feature of apoptosis, which involves the liberation of cytochrome from mitochondria. Additionally, initiator caspases and effector caspases also play a role in regulating this process (Bertheloot et al. 2021). A recent study has found that necrosis could also be regulated. Necroptosis, a form of controlled cellular death, takes place after the stimulation of tumor necrosis factor receptor 1 (TNFR1) by interacting with TNFα (Yan et al. 2022). The activation of inflammasome sensors leads to pyroptosis, which is marked by the disruption of the integrity of the plasma membrane. Inflammasomes function as a strong protective mechanism against pathogens or cellular pressure, resulting in destructive cell death that obstructs the proliferation of microorganisms and at the same time notifies the immune system of imminent dangers. However, dysregulated activation of this vital physiological sentinel function can also lead to the onset of pathological inflammation (Yan et al. 2022). Ferroptosis, a form of controlled cellular death, relies on iron and is distinguished by an overabundance of lipid peroxidation. It can be induced by IFNγ and TGFβ1 to suppress tumor progression (Chen et al. 2021). In contrast, cuproptosis entails the interaction of Cu^2+^ with lipoylated components of the tricarboxylic acid cycle in the respiratory chain of mitochondria (Chen et al. 2022a). The interaction causes the accumulation of proteins with lipoylation and the decrease of proteins with iron-sulfur clusters, leading to proteotoxic stress and eventual cell death. Entosis is a form of cell-in-cell-mediated death. Core elements essential for entotic cell-in-cell formation include contractile actomyosin, adherens junctions, and mechanical rings (Wang et al. 2020). Conversely, netotic cell demise is induced by the liberation of neutrophil extracellular traps (NETs), renowned mainly for their antimicrobial properties (Jiang et al. 2023). NETs have the function of capturing, restraining, and eradicating intruding bacteria and pathogens. The intricate extracellular formations are composed of nuclear DNA strands and mitochondria adorned with granular antimicrobial enzymes and histones. Different types of stress can cause lysosomal membrane permeabilization, leading to the movement of intralysosomal elements, like cathepsins, from the lysosomes to the cytoplasm (Wang et al. 2018). The commencement of lysosome-mediated cellular demise occurs through this procedure. Conversely, autophagy-triggered cellular demise takes place when autophagy is stimulated and acts as a safeguarding process to eliminate impaired cellular elements. Lately, alkaliptosis has surfaced as a pH-responsive type of controlled cellular death and is being investigated as an innovative approach for treating cancer (Liu et al. 2020). In contrast, oxeiptosis is a type of cellular death caused by reactive oxygen species and is propelled by the stimulation of the KEAP1-PGAM5-AIFM1 pathway. Previously, cell death pathways were believed to function independently without overlap. However, it is now evident that these pathways are intricately interconnected and can cross-regulate one another. Caspase-8, recognized as one of the earliest-discovered bridges between various cell deaths, inhibits the formation of necrosomes and promotes apoptosis over necroptosis (Wang et al. 2008). Additionally, the inactivation of procaspase-8 also promotes caspase-1-dependent cleavage of gasdermin D and pyroptosis (Newton et al. 2019). Thus, caspase-8 may be the key regulator among apoptosis, necroptosis, and pyroptosis.

Diverse PCD patterns have significant associations with tumorigenesis and development. The development of GBM, like other cancers, depends on avoiding various types of cell death. Nevertheless, the exploration of PCD’s role in GBM has been insufficient. Moreover, the prognosis and treatment response of GBM patients in relation to PCD-related genes have yet to be thoroughly analyzed. Hence, this research identified genes associated with survival and developed a novel indicator, the cell death index (CDI), to gauge the effectiveness of treatment for GBM patients. Differences in drug sensitivity and the tumor microenvironment between subgroups identified based on CDI were analyzed. In summary, our study constructed an accurate prognostic model to evaluate the survival outcomes of GBM patients and develop individualized and effective treatment strategies for them.

## Materials and Methods

### Data Collection

A total of 12 PCD pattern-related genes were identified, encompassing apoptosis (580 genes), necroptosis (101 genes), pyroptosis (52 genes), ferroptosis (87 genes), cuproptosis (14 genes), entotic cell death (15 genes), netotic cell death (8 genes), parthanatos (9 genes), lysosome-dependent cell death (220 genes), autophagy (367 genes), alkaliptosis (7 genes), and oxeiptosis (5 genes) (Tang et al. 2019; Zou et al. 2022). Forward analysis included a total of 1078 genes (Table S1).

The training group included transcriptome records from 159 GBM individuals acquired from the Cancer Genome Atlas (TCGA) repository (https://portal.gdc.cancer.gov/). Additionally, clinical data for these specimens was obtained. Data on copy number variation and masked somatic mutations were obtained from the GDC Data Portal (https://portal.gdc.cancer.gov/). To serve as a standard control, 30 normal tissues were obtained from the Genotype-Tissue Expression (GTEx) repository. The normalized expression matrix and corresponding clinical information of 210 GBM cases were acquired from GlioVis (http://gliovis.bioinfo.cnio.es/) for the microarray validation cohort. These samples were part of the REMBRANDT dataset. Furthermore, 135 GBM samples were obtained from the Chinese Glioma Genome Atlas (CGGA) portal (http://www.cgga.org.cn/index.jsp) (Zhao et al. 2017), along with transcriptome and clinical data. Given the population representative and data characteristics in these three public databases, we chose the database as the training cohort and the REMBRANT and CGGA databases as the validation databases. To ensure consistency in our study, only adult cases with complete clinical information were included. Samples lacking such information were excluded. Moreover, our attention was solely directed towards genes that exhibited expression levels greater than zero in over 50% of the samples.

### Identification of Key PCD-Related Genes Associated with GBM

The “limma” R package was used to identify differentially expressed genes (DEGs) between tumors and normal tissues in the TCGA cohort. The selection of DEGs was determined by a FDR that was adjusted to be below 0.05 and a logFC that exceeded 1 (Ritchie et al. 2015). The R package “clusterProfiler” was used to conduct Gene Ontology (GO) analyses with the DEGs. For investigating somatic mutation data within TNBC patients, the “maftools” package was used (Mayakonda et al. 2018). Values above 0.2 were considered “gains,” and values below − 0.2 were considered “losses” after filtering CNV values linked to programmed cell death (PCD)-related genes. Using the “circlize” R package (Gu et al. 2014), a circus plot was created to visually display the distinctive characteristics of the PCD-related genes.

### Prognostic Model Construction and Validation

PCD-associated DEGs were regarded as potential prognostic genes if they showed significance (*p* < 0.05) in both the Kaplan–Meier and univariate Cox analyses. The genes underwent a LASSO Cox analysis using the R package “glmnet” to determine the optimal penalty parameter lambda. The examination was conducted in the TCGA-GBM group (Friedman et al. 2010). The CDI, which stands for coefficient-based gene expression index, was computed by utilizing the normalized levels of gene expression and their corresponding regression coefficients. The formula used for calculating CDI is as follows: CDI = sum (gene expression level × corresponding coefficient). In the training cohort, the threshold value for CDI was determined using the X-tile program (Camp et al. 2004 Nov 1). Based on this threshold, the GBM patients were categorized into two subgroups: low CDI and high CDI. To investigate the variation in gene expression patterns among GBM samples, principal component analysis (PCA) was performed using the “stats” package. Moreover, an examination of Kaplan–Meier was performed to evaluate the correlation between the duration of overall survival (OS) and CDI. The examination employed the R packages “survival” and “survminer” for analysis.

### Functional Enrichment Analysis

The “clusterProfiler” R package was used to perform gene set enrichment analysis (GSEA). The examination employed the collection of gene sets known as “c2.all.v7.0.entrez.gmt,” sourced from the Molecular Signatures Database (MSigDB) (Hänzelmann et al. 2013). This database contains a comprehensive collection of gene sets associated with various biological pathways and processes.

### Unsupervised Clustering Analysis in GBM

Consensus clustering analysis was conducted using the R package “ConsensusClusterPlus” to discover unrecognized subtypes of GBM utilizing 14 genes associated with PCD (Wilkerson and Hayes 2010). The CC analysis involved setting the parameter “maxK” to 10, selecting “clusterAlg” as “km” (k-means), and using “distance” as “pearson” for calculating the similarity between samples. The TCGA-GBM cohort was utilized as the training set in this analysis, whereas the REMBRANDT and CGGA datasets were employed to assess the reliability of the clustering outcomes.

### Nomogram Development and Evaluation of Predictive Performance

Univariate and multivariate Cox regression analyses were conducted in the TCGA-GBM cohort to determine independent prognostic factors, utilizing both the patient’s clinical information and CDI. These analyses aimed to determine which variables significantly influenced overall survival. To visually represent the prognostic model, a nomogram plot was generated using the “regplot” package. This plot provides a visual tool for estimating individualized survival probabilities based on the identified prognostic factors. The effectiveness of the predictive model was assessed using calibration plots and decision curve analysis (DCA), employing the R software packages “caret” and “rmda.” Calibration graphs evaluate the concordance between the observed and estimated chances of survival. The clinical utility of the model is evaluated by DCA, which measures the overall advantage at various threshold probabilities. The “timeROC” R package was used to conduct receiver operating characteristic (ROC) analyses. These analyses assess the discriminatory power of the prognostic model in predicting survival outcomes.

### Drug Sensitivity and the Tumor Immune Microenvironment Analysis

To forecast drug sensitivities between the CDI-high and CDI-low groups (Ohashi et al. 2020), the R package “oncoPredict” was employed. This analysis aimed to assess potential differences in drug responses between these two subgroups based on their CDI values. The immune microenvironment was assessed by determining the infiltration scores of 28 immune cells using single-sample gene set enrichment analysis (ssGSEA) through the utilization of the “gsva” R package (Mariathasan et al. 2018). The analysis offers a calculation of the proportionate prevalence of various immune cell categories in the tumor specimen. Moreover, an analysis was conducted on the correlation between CDI and immunomodulatory agents. This analysis aimed to investigate potential associations between CDI values and genes involved in immune regulation or modulation.

### Single-Cell RNA-Seq Data Processing and Analysis

We analyzed the RNA-seq data of GBM patients’ tumor tissues, which were obtained from GSE162631, using the R package “Seurat” (Hao et al. 2021). Assessing the proportion of genes related to mitochondria or ribosomes was used to evaluate the quality of the initial counts. Cells with low-quality counts were removed from the analysis. To identify the top 2000 genes that exhibit high variability, the Seurat package’s “FindVariableFeatures” function was utilized. The “FindNeighbors” and “FindClusters” functions were used to analyze cell clustering. The “FindNeighbors” function was used to create a k-nearest neighbor graph in principal component analysis (PCA), which relied on Euclidean distance to identify the closest neighbors for every cell. Using the “RunTSNE” function, t-distributed stochastic neighbor embedding (tSNE) was then applied. The cell clusters were visualized using the resulting dimensions of tSNE-1 and tSNE-2. For each cluster, the “FindAllMarkers” function was utilized to perform differential expression analysis, identifying marker genes based on a cutoff threshold where the adjusted *p*-value was below 0.01 and the absolute log2 fold change exceeded 1. The “Garnett” package was employed for clustering annotations to identify distinct cell types (Pliner et al. 2019). The “analyze_sc_clusters” function was used for enrichment analysis, and the results were extracted based on pathways. The “AddModuleScore” function was utilized to calculate module scores and the proportion of enrichment for gene expression related to PCD in individual cells. The “ReactomeGSA” package (Griss et al. 2020) was used to perform functional enrichment analysis on the identified hub cell types. The differentiation trajectories between various developmental stages were captured using CytoTRACE, with cells exhibiting the highest CytoTRACE score indicating immaturity or an early stage of development (Gulati et al. 2020). Finally, cell–cell communication analysis and network visualization were carried out using the “CellChat” and “patchwork” software packages, respectively (Jin et al. 2021). The purpose of these analyses was to investigate communication patterns and create a visual representation of the network of interactions among various cell types.

### Statistical Analysis

R software, version 4.2.2, was utilized for conducting all statistical analyses. To analyze disparities between two groups, either the Student's t-test or the Wilcoxon test was utilized, depending on the characteristics of the data. The Kruskal–Wallis test was employed for making comparisons between multiple groups. We performed survival analysis by utilizing Kaplan–Meier diagrams and evaluated disparities between survival curves through the log-rank test. For all analyses, a statistical significance level of P < 0.05 was deemed significant.

## Results

**Fig. 1:**
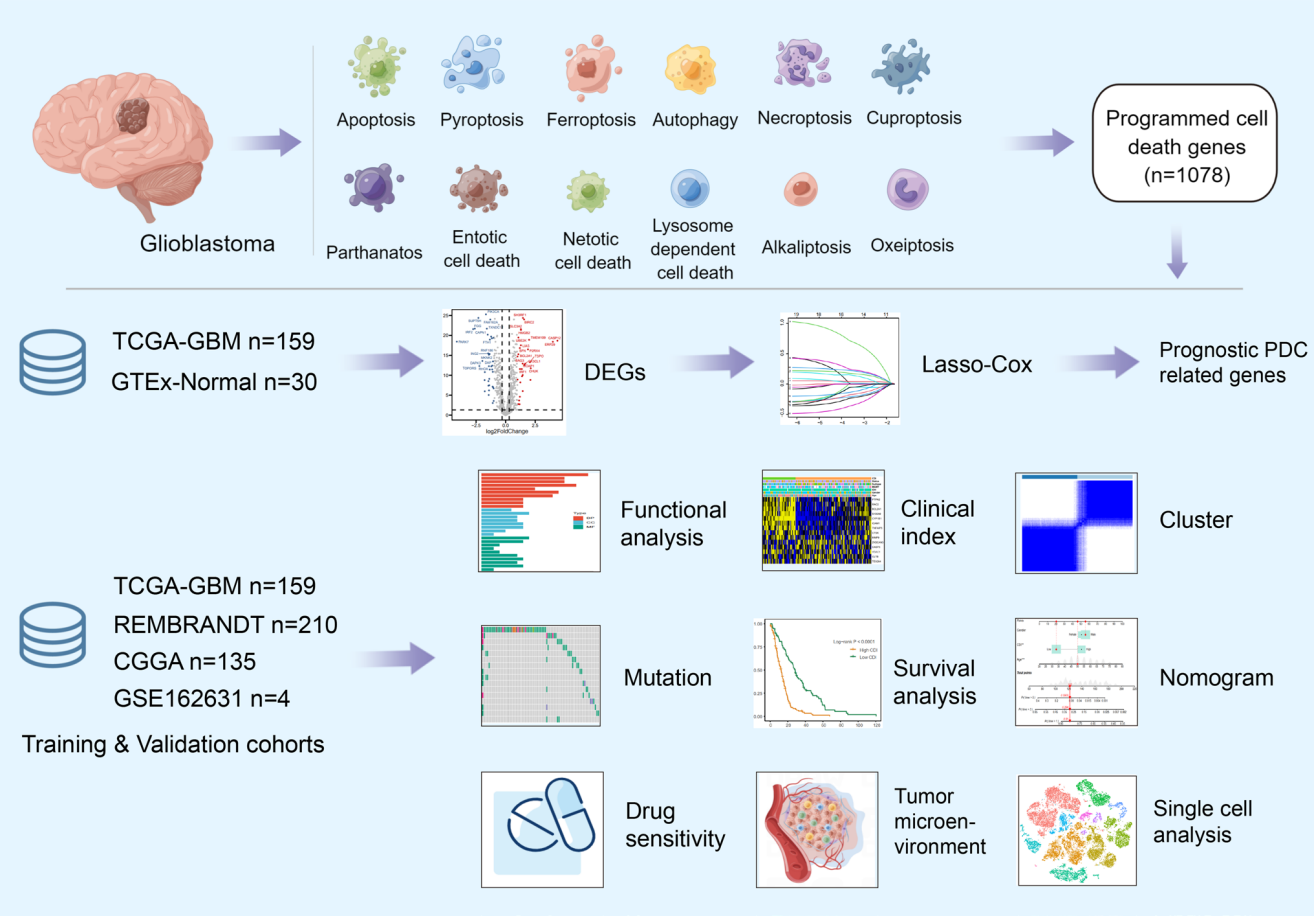
Flowchart for comprehensive analysis of 11 programmed cell death (PCD) patterns in GBM patients

The TCGA-GBM database was selected as the training cohort, while the REMBRANT and CGGA databases were chosen as the validation cohorts for the prognosis predictive model. Transcriptional heterogeneity was evaluated using single-cell RNA-seq data (GSE162631) obtained from four patients with GBM. The detailed process of this study is illustrated in Fig. 1.

### Differential Expression of PDC-Related Genes in GBM Patients

**Fig. 2:**
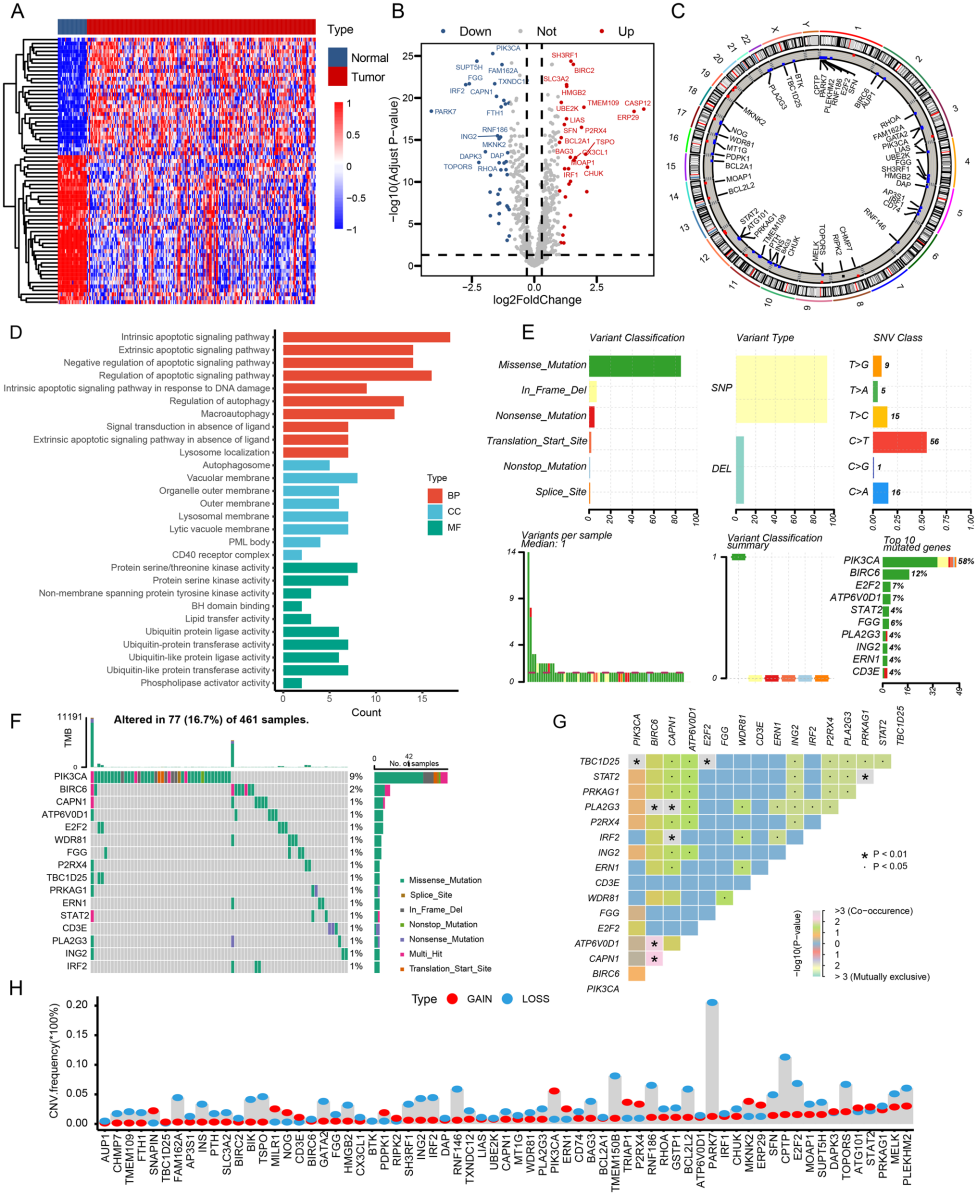
Differential expression of PDC-related genes in GBM Patients. **A**, **B** Heatmap and volcano plot of the PDC-related DEGs between GBM and normal tissues. **C** The location, expression, and correlation of PCD-related differential expression genes (DGEs) in the TCGA cohort. **D** GO enrichment analyses based on the DEGs. **E**, **F** An oncoplot of PCD-related genes in the TCGA cohort. **G** The correlogram of interactions between highly mutated PCD-related genes. **H** CNV values of PCD-related genes in the TCGA cohort

A total of 68 differentially expressed genes (DEGs) were identified in the TCGA-GBM dataset. The differentially expressed genes (DEGs) exhibited a *p*-value below 0.05 after adjustment and a fold change exceeding 1 on the absolute log2 scale. Out of these, the GBM group exhibited upregulation of 30 genes, whereas 38 genes showed downregulation. The heatmaps in Fig. 2A display the scaled RNA levels of the differentially expressed genes (DEGs). Furthermore, Fig. 2B showcases a volcano plot that emphasizes the differentially expressed genes (DEGs). For more in-depth details on the chromosomal position, expression trends, and associations of every DEG, refer to Fig. 2C. Moreover, the GO enrichment analysis revealed several biological pathways associated with these differentially expressed genes, as depicted in Fig. 2D. Furthermore, the examination evaluated the diversity of genes associated with PCD among patients with GBM in the TCGA group. The results revealed that approximately 16.7% of GBM patients exhibited mutations in PCD-related genes. In Fig. 2E, the top ten mutations of these genes are shown, with PIK3CA having the highest frequency of mutations (58%), followed by nine other genes ranging from 12 to 4% (Fig. 2F). Figure 2G depicts the connections among extensively mutated genes associated with PCD. Furthermore, the analysis of CNV status revealed frequent changes in genes associated with PCD. In particular, PARK7 displayed the highest occurrence of CNV deletion, while YPIK3CA demonstrated notable amplifications in copy number (Fig. 2H).

### Prognostic Signature Construction with PCD-Related Genes

**Fig. 3:**
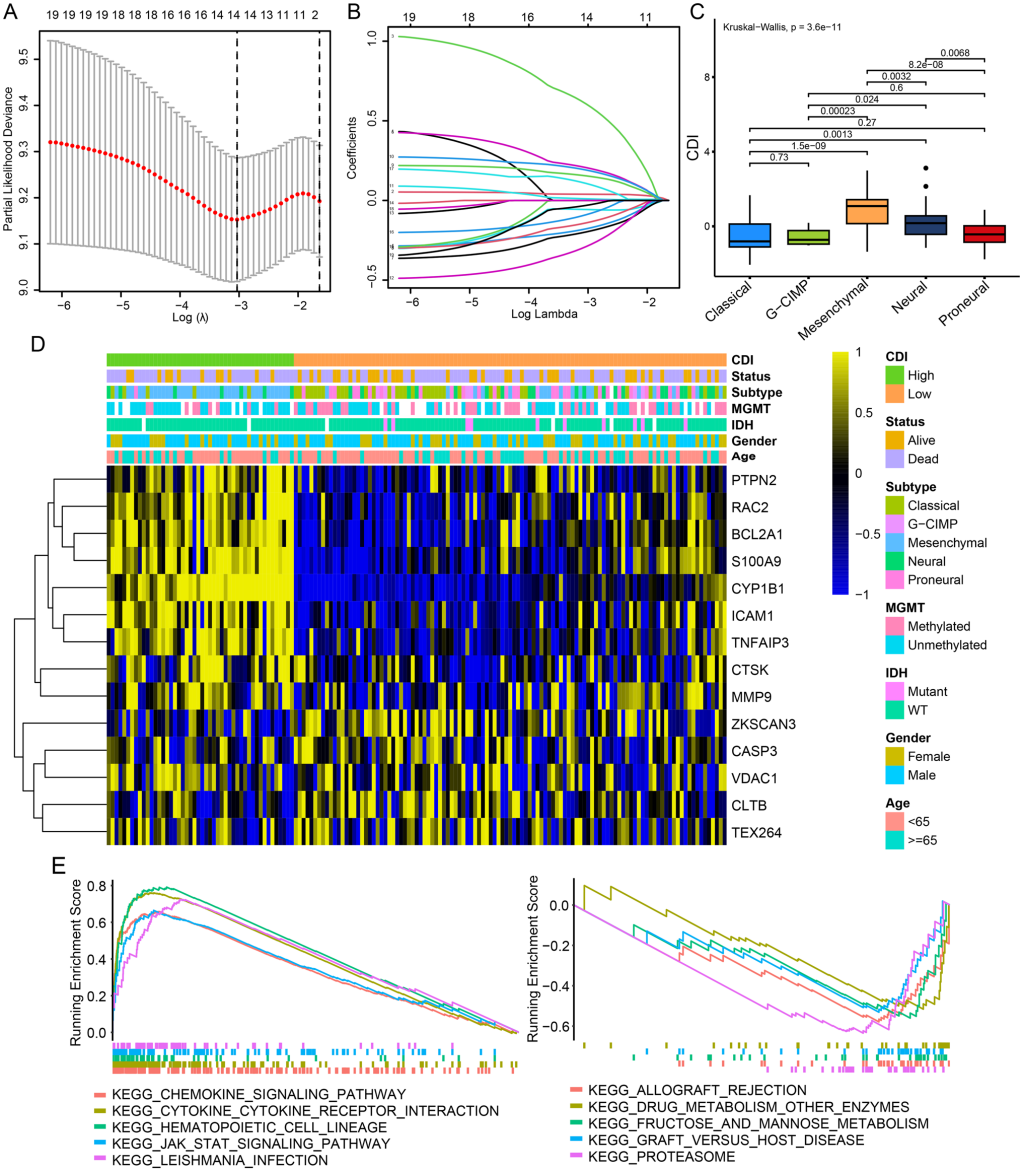
Prognostic signature construction with PCD-related genes. **A**, **B** Identification of the 14 model genes by LASSO regression analysis. **C** Violin plots of the relationship between CDI and different GBM molecular subtypes. **D** Heatmap of 12 model genes and clinical features. **E** Pathway enrichment analysis between high- and low-CDI groups by GSEA

Survival data from GBM patients was collected and subjected to analysis. At first, a univariate Cox regression analysis was used to discover 19 genes linked to survival. Figure S1 demonstrates the further validation of the results through Kaplan–Meier analysis. LASSO Cox regression analysis effectively reduced features in high-dimensional data and optimized predictors of clinical outcomes. The vertical dotted line in Fig. 3A corresponds to the penalty value of the lowest point (that is, the upper coordinate corresponding to the lowest point of the curve). Then, we find the vertical line corresponding to the corresponding position of the penalty value in Fig. 3B. The number of intersecting points is the number of variables included in the final model, and the ordinate of the corresponding intersection point is the regression coefficient of the variable. We took the value of log(λ) to − 3, and 14 genes were finally selected from 19 genes. Among these genes, there were eight genes associated with apoptosis, five genes linked to autophagy, three genes connected to lysosome-dependent cell death, two genes related to necroptosis, and one gene associated with pyroptosis. Figure S2 illustrates the examination and representation of the association among every gene in the model. Moreover, the levels of expression for these 14 genes were compared between TNBC tissues and normal samples using the Wilcoxon test (Figure S3). Significant differences (*p* < 0.05) were detected in the expression of all 14 genes. The hazard model was constructed by combining the 14 PCD-associated genes using their coefficients from the multivariate Cox analysis, according to the formula:

$$\begin{aligned}CDI=\left(E_{\mathrm{BCL}2\mathrm A1}\times0.15\right)+\left(E_{\mathrm{CASP}3}\times-0.05\right)+\left(E_{\mathrm{CLTB}}\times0.14\right)\\+\left(E_{\mathrm{CTSK}}\times-0.19\right)+\left(E_{\mathrm{CYP}1\mathrm B1}\times0.60\right)+{(E}_{\mathrm{ICAM}1}\times-0.03)\\+(E_{\mathrm{MMP}9}\times0.03)+(E_{\mathrm{PTPN}2}\times-0.29)+(E_{\mathrm{RAC}2}\times-0.09)\\+(E_{\mathrm S100\mathrm A9}\times0.19)+(E_{\mathrm{TEX}264}\times0.01)+(E_{\mathrm{TNFAIP}3}\times0.11)\\+(E_{\mathrm{VDAC}1}\times-0.18)+(E_{\mathrm{ZKSCAN}3}\times0.20)\end{aligned}$$

*E*_BCL2A1_ represents the expression value of the gene BCL2A1, while the remaining genes have a similar representation to BCL2A1. Using the X-tile program, a threshold value of 1.58 was determined for CDI. Using this criterion, the TCGA cohort was segregated into a high-CDI group and a low-CDI group, comprising 159 GBM patients, which were then utilized as the training dataset. The presence of CDI was found to have a significant association with various clinical characteristics, such as distinct molecular subtypes of GBM (as shown in Fig. 3C). Heatmaps were employed to illustrate the associations between CDI and clinical characteristics, along with the status of survival. However, no significant relationship was observed (Fig. 3D). In order to further examine variations in biological processes among the subgroups classified by the gene signature, the application of gene set enrichment analysis (GSEA) was utilized. In the TCGA cohort, Fig. 3E showcases the top ten biological processes that have been identified.

### Internal Training and External Validation of the PCD-Related Signature

**Fig. 4:**
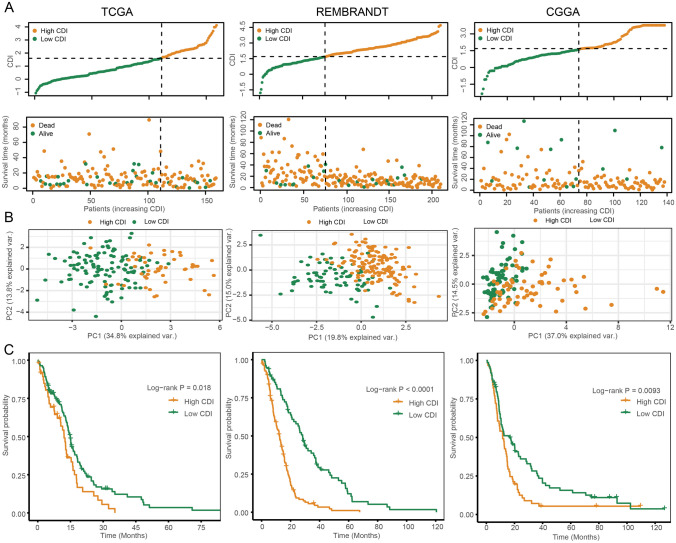
Internal training and external validation of the PCD-related signature. **A** Distribution of adjusted CDI according to the survival status and time in TCGA, REMBRANDT, and CGGA cohorts. **B** Principal component analysis (PCA) plot based on the CDI in TCGA, REMBRANDT, and CGGA cohorts. **C** Overall survival in the low- and high-CDI group patients in TCGA, REMBRANDT, and CGGA cohorts

The results of our study showed that in the TCGA-GBM cohort, consisting of 159 patients, which served as the internal training dataset, as well as in the external validation cohorts REMBRANDT (210 patients) and CGGA (135 patients), individuals with elevated CDI levels (> 1.58) exhibited a decreased rate of survival in comparison to those with lower CDI levels (≤ 1.58) (as depicted in Fig. 4A). PCA was conducted to assess the categorization using CDI, and the findings demonstrated a favorable classification, as depicted in Fig. 4B. Furthermore, a significant distinction was noted in the duration of overall survival (OS) between the two groups with CDI. In particular, individuals in the low-CDI category demonstrated a greater likelihood of encountering reduced mortality rates (*p* < 0.05, as shown in Fig. 4C). Additionally, a connection was established between the mutation status and the CDI groups. Significantly, our results indicated a higher occurrence of PTEN mutations in patients belonging to the high-CDI group (*p* < 0.05, Figure S4).

### Unsupervised Clustering of PCD-Related Model Genes

**Fig. 5:**
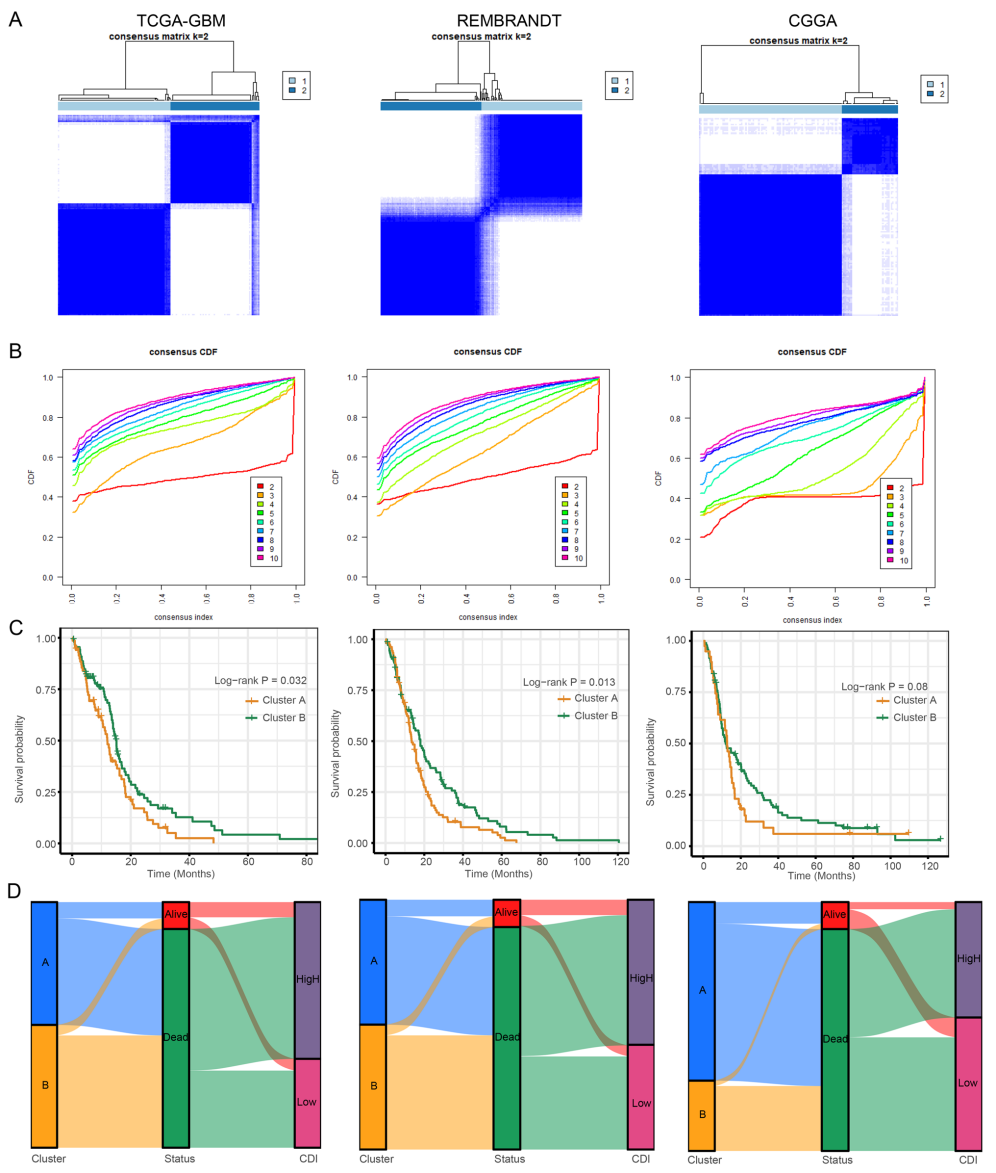
Unsupervised clustering of PCD-related model genes. **A** GBM patients were grouped into two molecular clusters when *k* = 2, based on the PCD-related model genes. **B** Representative cumulative distribution function (CDF) curves. **C** Kaplan–Meier survival curve analysis between the two clusters. **D** Alluvial diagram shows the interrelationship between molecular clusters, survival status, and CDI groups in GBM patients

A comprehensive analysis was carried out to investigate unknown variations of GBM, utilizing a collection of 14 genes associated with PCD. Significantly, the subgroups exhibited the most noticeable disparities when the value of *k* (number of clusters) was established as 2. The implication was that the TCGA-GBM cohort, consisting of 159 patients, could be successfully categorized into two separate groups, as shown in Fig. 5A and B. Moreover, there was a notable difference in the duration of overall survival (OS) time among these clusters, with a *p*-value of 0.032 (Fig. 5C). Cluster B exhibited a more favorable prognosis, while cluster A was characterized by a poorer prognosis. Similar results were also observed in the REMBRANDT dataset (*p*-value = 0.013) and the CGGA dataset (*p*-value = 0.081). Furthermore, the clusters were visualized using alluvial diagrams to depict the distribution patterns of CDI. Figure 5D shows that most patients in cluster A had elevated CDI values, while a significant number of patients in cluster B had decreased CDI values.

### Construction and Assessment of the Nomogram Survival Model

**Fig. 6:**
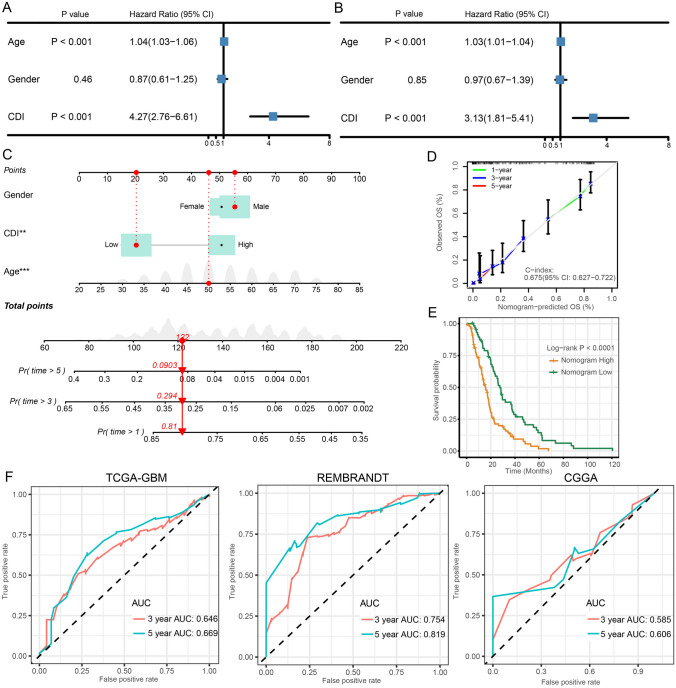
Construction and assessment of the nomogram survival model. **A**, **B** Univariate and multivariate analysis of CDI and the clinicopathologic characteristics. **C** A nomogram was established to predict the prognostic of GBM patients. **D** The calibration curve of the nomogram. **E** Kaplan–Meier analyses for the two GBM groups based on the nomogram score. **F** Receiver operator characteristic (ROC) analysis of nomogram in TCGA, REMBRANDT, and CGGA cohorts

To ascertain whether CDI could serve as a prognostic factor independently, both univariate and multivariate Cox regression analyses were conducted. According to the univariate analysis findings, CDI was found to be a significant risk factor in comparison to other factors (HR = 4.27, 95% CI 2.76–6.61, *p* < 0.001, Fig. 6A). Furthermore, even after accounting for other influencing factors, the multivariate analysis revealed that CDI continued to be a separate predictive factor in GBM patients (HR = 3.13, 95% CI 1.81–5.41, *p* < 0.001, Fig. 6B). A nomogram model was created in the TCGA cohort using multivariable Cox regression and stepwise regression analyses to predict the overall survival (OS) for 1, 3, and 5 years. Figure 6C includes gender and CDI as predictor variables in the nomogram. The model’s predictive accuracy was calculated as 0.675 (95% CI 0.627–0.722), as indicated by its *C*-index value. To evaluate the precision of the model in forecasting the survival rates for 1, 3, and 5 years, calibration curves were created (Fig. 6D). Significantly, there was a clear contrast in survival rates between the high and low groups, as indicated by the nomogram score (Fig. 6E). In addition, the AUC values were assessed in three separate groups, indicating that the nomogram showed excellent precision in forecasting the survival of GBM patients for both 3 and 5 years (Fig. 6F).

### Drug Sensitivity Analysis and Landscape of the Immune Microenvironment

**Fig. 7:**
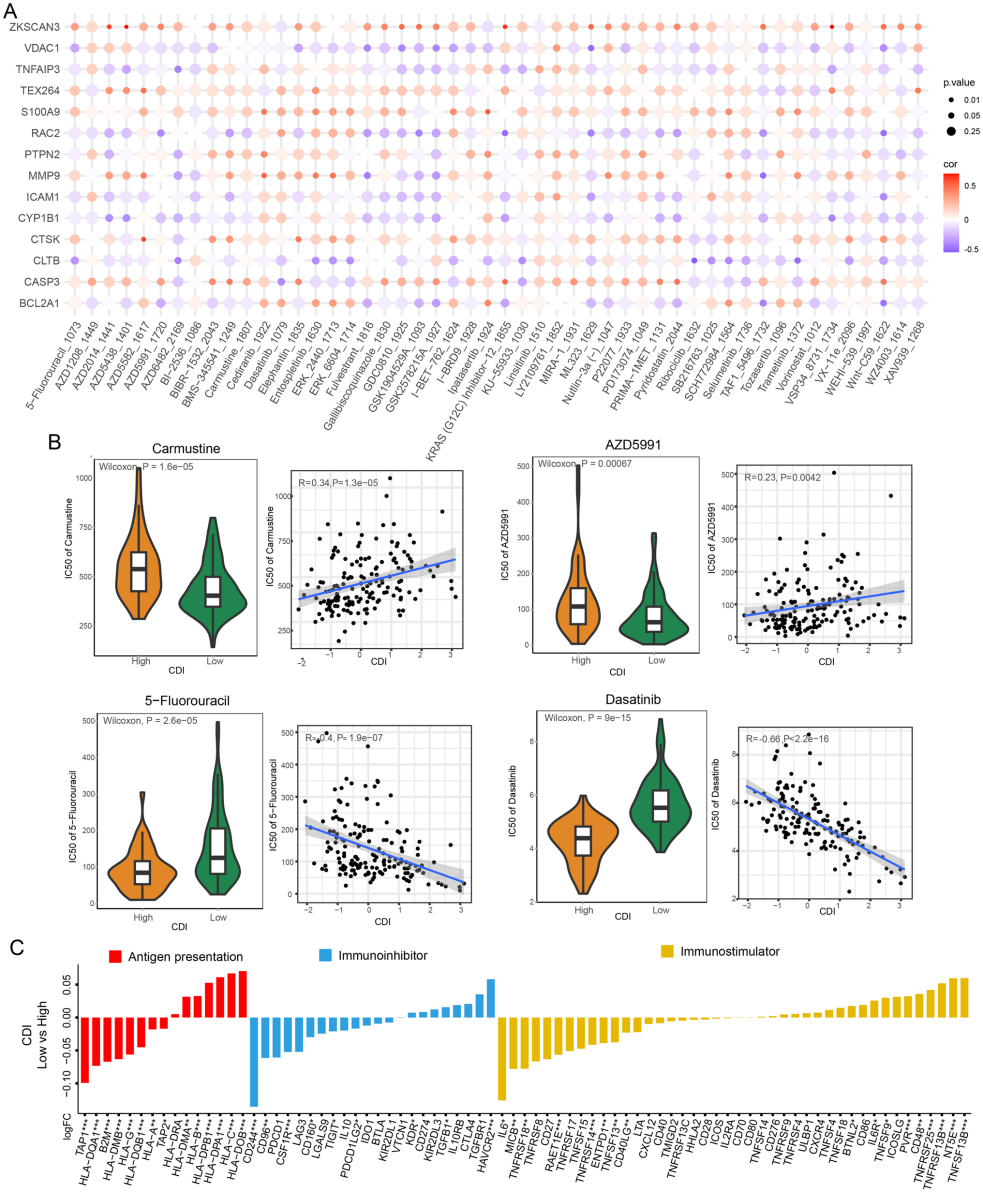
Drug sensitivity analysis and landscape of the immune microenvironment. **A** Bubble plot of the correlation between drugs and model genes. **B** Boxplots of IC50 of carmustine, AZD5991, 5-fluorouracil, and dasatinib in high- and low-CDI groups. **C** Bar plot of the correlation between immunomodulators and the CDI values in GBM patients

In order to examine the correlation between the established model and drug responsiveness, we computed the IC50 values, representing the concentration at which half of the drug’s inhibitory effect is achieved, for every GBM sample to detect noteworthy variances. Figure 7A and B illustrate the relationship and importance of drug sensitivities and CDI. Surprisingly, the group with high CDI demonstrated increased IC50 values for carmustine and AZD5991, suggesting a possible resistance to these medications. Conversely, 5-fluorouracil and dasatinib exhibited lower IC50 values, suggesting a possible susceptibility to these medications. The results indicate that individuals with elevated CDI levels in GBM may exhibit resistance to carmustine but could potentially show sensitivity to 5-fluorouracil. Consequently, 5-fluorouracil shows promise as a treatment option for chemotherapy-resistant GBM patients with high CDI.

Furthermore, we performed an examination to discern disparities in immunomodulating agents and cells associated with the immune system among the two groups with CDI. According to the bar graph shown in Fig. 7C, it can be inferred that the low-CDI group might demonstrate more robust immune responses. In addition, the enrichment scores for immune-related cells were computed, revealing that only consistently activated NK cells exhibited significantly elevated levels in the low-CDI group across the three cohorts. Figure S5 demonstrates a notable positive correlation between CDI and the increased activation of these elevated levels of NK cells.

### Single-Cell RNA Transcriptome Data in GBM

**Fig. 8:**
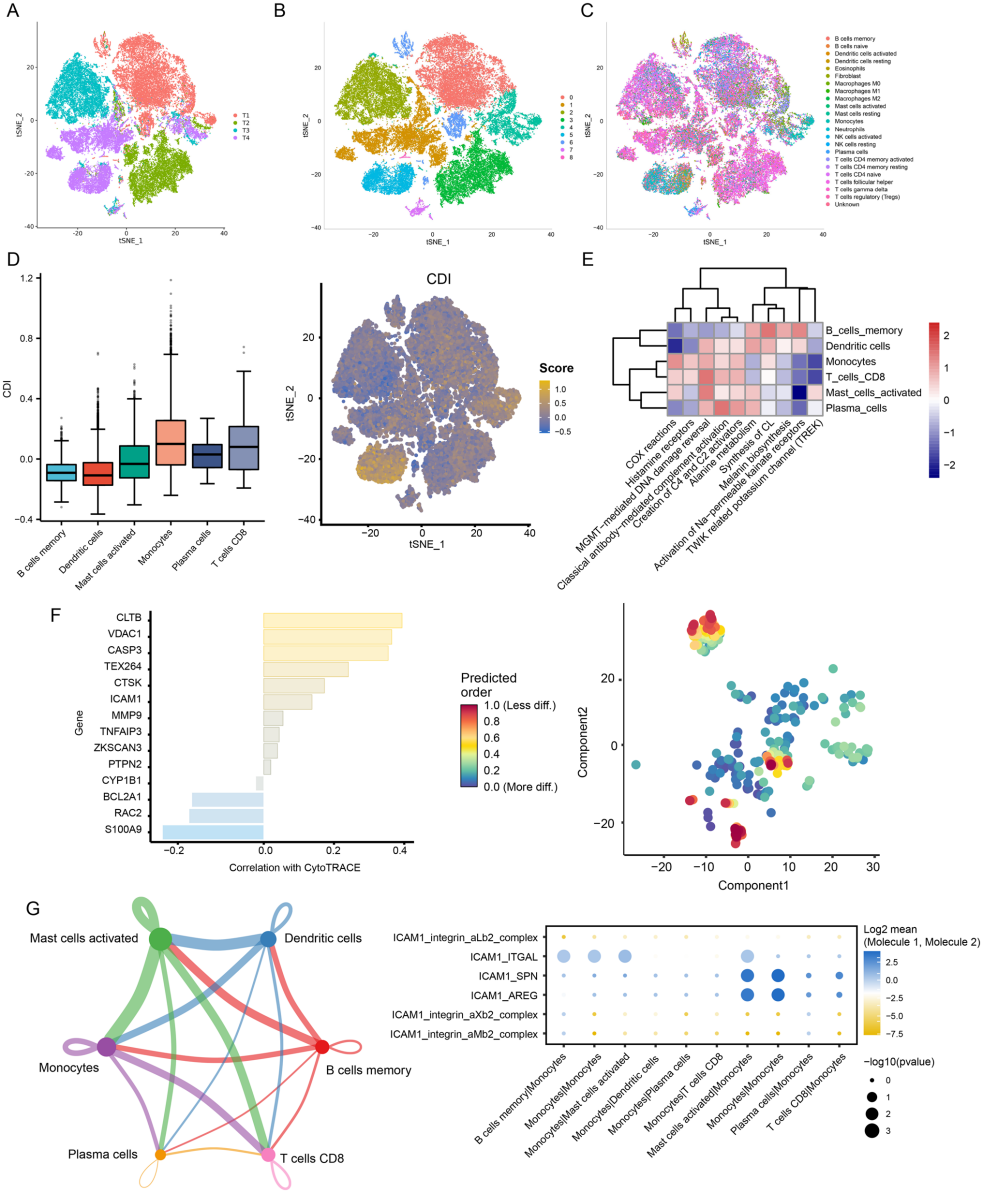
Single-cell RNA transcriptome data analysis in GBM. **A**–**C** Cluster annotation and cell type identification by tSNE. **D** Boxplots showing CDI of six subtypes of cell in GBM microenvironment and feature plot showing CDI of monocytes. **E** Functional enrichment analysis of identified six cell types. **F** Correlation between 14 PCD-related genes and CytoTRACE score and a predicted higher developmental potential for monocytes (cells with the highest CytoTRACE score mapped to the starting point of the trajectory by RNA velocity). **G** Among the six cell types, there were high cell-to-cell correlations in terms of the number and intensity of ligand-receptor interactions

To acquire a more comprehensive comprehension of the distribution of CDI in GBM patients, we examined data from the single-cell RNA transcriptome (GSE162631). Following the process of quality control (as shown in Figure S6A-B), the cells underwent clustering using the FindNeighbors and FindClusters functions, with a resolution of 0.1, which led to the formation of nine distinct clusters (as depicted in Figs. 8A and B and S6C). Figure S6D displays the top five marker genes for every cell type. Using the Garnett package, we performed cluster annotation and tSNE visualization of downscaled cell types, identifying 22 known cell types (Fig. 8C). In the GBM microenvironment, it was noticed that monocytes displayed higher CDI values compared to other cell types among these cell types (Fig. 8D). The ReactomeGSA analysis showed that COX reactions, histamine receptors, MGMT-mediated DNA damage reversal, and classical antibody-mediated complement activation pathways were mainly associated with memory B cells, dendritic cells, monocytes, CD8 + T cells, activated mast cells, and plasma cells (Fig. 8E). Moreover, we examined the association between the 14 genes associated with PCD and the CytoTRACE score, which forecasts the developmental capacity of a cell. According to this analysis (Fig. 8F), monocytes exhibited a greater capacity for development. Furthermore, by employing RNA velocity, the cells exhibiting the greatest CytoTRACE score, which signifies the least mature cells, were assigned to the initial point of the trajectory. In order to further explore the communication between cells, we examined the connection among monocytes and memory B cells, dendritic cells, CD8 + T cells, activated mast cells, and plasma cells. Significantly, the involvement of ICAM1-ITGAL, ICAM1-SPN, and ICAM1-AREG in monocytes was observed to have pivotal functions in ligand-receptor interactions (Fig. 8G).

## Discussion

The present research is the initial extensive examination of 12 different programmed cell death (PCD) patterns in GBM. The process included building a signature for cell death in the TCGA-GBM group, which was later confirmed by two additional groups (REMBRANDT and CGGA), showcasing its outstanding effectiveness. Additionally, a nomogram model that incorporated clinical characteristics and CDI was developed, resulting in favorable outcomes for predicting overall survival. Moreover, this research examined the possible association between CDI and immunomodulating agents, the tumor microenvironment, and the sensitivity of drugs. The analysis uncovered connections between CDI and immune-related cells, indicating variations in immune functions among the high- and low-CDI groups. Additionally, the investigation examined the correlation between CDI and drug responsiveness, uncovering possible resistance to carmustine while revealing potential susceptibility to 5-fluorouracil among patients with high CDI. In general, this research offers an extensive understanding of the PCD patterns in GBM and their significance for prognosis, immune response, tumor microenvironment, and drug sensitivity.

PCD is a multifaceted phenomenon governed by diverse mechanisms. Accumulating evidence substantiates the pivotal involvement of PCD in fundamental biological processes and its long-standing association with the onset and spread of malignant neoplasms (Su et al. 2015). In our study, we developed a signature comprising 14 PCD-related genes (BCL2A1, CASP3, CLTB, CTSK, CYP1B1, ICAM1, MMP9, PTPN2, RAC2, S100A9, TEX264, TNFAIP3, VDAC1, and ZKSCAN3) and demonstrated its ability to predict overall survival in GBM patients. BCL2-related protein A1 (BCL2A1), an antiapoptotic protein, promotes anti-cancer drug resistance (Li et al. 2021). The presence of excessive BCL2A1 levels was detected and linked to unfavorable survival outcomes in GBM. However, BCL2A1 is a potential target in cancer therapy, and inhibitors of this protein are developing. Caspase 3 (CASP3) was found to be involved in apoptosis, pyroptosis, and autophagy pathways at the same time in GBM in our study. A favorable prognosis was correlated with a relatively heightened expression of CASP3. A recent investigation has indeed validated that the inhibition of glioblastoma growth is caused by CASP3, which triggers intrinsic apoptosis and pyroptosis by means of the mediated cleavage of gasdermin E (Zhao et al. 2023). Currently, there is limited mechanism investigation of clathrin light chain B (CLTB) in cancer. According to reports, CLTB is said to be involved in the invasion and spread of tumors (Mukenhirn et al. 2021). Cathepsin K (CTSK), a cysteine protease, plays a significant role in tissue invasion and angiogenesis within glioblastoma multiforme (GBM) (Santangelo et al. 2021). It is worth mentioning that the presence of CYP1B1 has been solely detected in medulloblastoma, distinguishing it from other types of central nervous system tumors. Previous studies have demonstrated that an immunotherapeutic agent designed to target this antigen can effectively and safely induce a robust immune response (Barnett et al. 2007). Our investigation revealed an upregulation of CYP1B1 in samples of glioblastoma multiforme (GBM). Furthermore, a relatively high level of cyclin-dependent kinase inhibitor 1A (CDKN1A) expression was discovered to have a strong association with an adverse prognosis. Intercellular adhesion molecule 1 (ICAM1) was observed to serve as a pivotal communicator between tumors and macrophages, influencing the GBM invasion (Yoo et al. 2022). ICAM1 is also involved in bevacizumab resistance in GBM (Piao et al. 2017). High expression of matrix metallopeptidase 9 (MMP9) in tumors is closely related to invasion, while a high plasmatic MMP9 level is associated with non-benefit from bevacizumab treatment (Jiguet-Jiglaire et al. 2022). GBM patients exhibiting elevated levels of MMP9 in tumor tissue or peripheral blood exhibited a notably poorer survival outcome, which is consistent with our results. PTPN2, a non-receptor type 2 protein tyrosine phosphatase, plays a vital role in both the development of tumors and the field of cancer immunotherapy as an immune checkpoint. Notably, the inhibition of PTPN2 has been observed to impede the proliferation of glioblastoma multiforme (GBM) (Tang et al. 2023). GBM exhibited a significant upregulation of Rac family small GTPase 2 (RAC2), which was found to be correlated with unfavorable survival outcomes. Paracrine factor S100 calcium-binding protein A9 (S100A9) could activate prosurvival factors Erk1/2 and p70S60k to promote GBM development (Hu et al. xxxx). Testis-expressed 264, the ER-Phagy receptor (TEX264), was reported to be involved in restoring cellular energy levels and endoplasmic reticulum homeostasis in GBM through autophagy (Zielke et al. 2021). Activation of the Akt and NF-κB signaling pathways may lead to temozolomide resistance in GBM by inhibiting TNF-alpha-induced protein 3 (TNFAIP3), as suggested by Chen et al. 2022b. The initiation of reprogramming malignant cancer cells into terminally differentiated cells, resulting in the reversal of their oncogenic properties within GBM, was triggered by the depletion of voltage-dependent anion channel 1 (VDAC1) (Arif et al. 2017). Zinc finger containing KRAB and SCAN domains 3 (ZKSCAN3) was discovered to enhance tumor migration in different types of cancers, such as breast, colorectal, and prostate (Chi et al. 2018; Cho et al. 2022; Zhang et al. 2012), except for GBM. An elevated expression of ZKSCAN3 was detected in GBM tissue samples, exhibiting a significant correlation with an unfavorable prognosis.

Analysis of drug sensitivity indicated that individuals in the high-CDI category displayed increased IC50 values for carmustine and AZD5991, suggesting a lack of responsiveness to these medications in the treatment of TNBC. On the other hand, the group with high CDI showed decreased IC50 values for 5-fluorouracil, indicating possible advantages of using this chemotherapy medication for GBM patients with CDI. Furthermore, individuals with elevated CDI exhibited the highest responsiveness to dasatinib, necessitating additional exploration via clinical trials to determine its effectiveness as a potential therapy for GBM with high CDI. Despite the significant progress made by immune checkpoint inhibitors (ICIs) in immunotherapy for cancer, only a minority of patients (20%) respond positively to ICIs (Zhang and Zhang 2020). According to prior studies, the presence of immune cells infiltrating the tumor (Zhang and Zhang 2020) can impact the prognosis of tumor patients and the efficacy of immunotherapy. The main reliance of current immune checkpoint inhibitors (ICIs), like PD1/PDL1 inhibitors, in clinical applications is on the activation of adaptive immunity mediated by T cells to eradicate cancer cells. However, this treatment approach has limited efficacy in GBM. The effectiveness of activating natural immune cells relies on the existence of functional T cells in the tumor microenvironment. Tumor types with higher mutational loads tend to exhibit greater infiltration of T cells, whereas those with lower mutational loads, like GBM, typically have lower levels of T cell infiltration (Nakamura and Smyth 2020). In all three cohorts, our research discovered a notable decrease in the number of natural killer (NK) cells present in the tumor microenvironment among the high-CDI subgroup. There was no observed variation in the distribution of CD8 + T cells. Regarding the identification and removal phase of the cancer-immunity cycle, natural killer (NK) cells have a wider array of receptors for detecting tumor cells that do not exhibit MHC/human leukocyte antigen (HLA) expression. Furthermore, they have the ability to swiftly eradicate atypical cells without the need for T cells (Wang et al. 2022). Furthermore, NK cell-induced apoptosis of tumor cells leads to an increased release of cancer cell antigens. Therefore, it is crucial to explore NK cells as potent immune modulators against tumors and conduct additional research on their role in GBM. Through the analysis of single-cell RNA transcriptome data, it was discovered that monocytes exhibited the greatest CDI in GBM. The analysis of bulk RNA transcriptome data in CGGA revealed a notable upsurge in the abundance of monocytes. The majority of immune cells that infiltrate GBM are macrophages and monocytes, and these cells are believed to have protumor and immunosuppressive effects in GBM (Tomaszewski et al. 2019). A prior investigation showed that the overexpression of CXCL1 and CXCL2 by interleukin 6 can enhance the recruitment of monocytes and drive macrophages towards a protumor phenotype, ultimately leading to the formation of a suppressive tumor microenvironment (Yeung et al. 2013). This discovery mentioned above might clarify the reason behind the unfavorable prognosis observed in patients belonging to the high-CDI category.

Although our CDI-derived model showed promising results in both the training and validation groups, there are various constraints to consider in this study. Initially, the retrospective analysis utilized data from a public database to identify clusters, develop prognostic models, and validate them. Hence, it is crucial to gather empirical data and verify the practicality of our forecasting model. Despite our efforts to address this issue through multivariate Cox regression analyses, the potential impact on the efficacy of our prediction model may have been influenced by the limited inclusion of comprehensive clinical characteristics in public databases. Noting these limitations is crucial since they emphasize the necessity of further research to collect more data and confirm the effectiveness of our model in a real-life scenario. In addition, this research involved a restricted set of genes associated with parthanatosis, netotic cell death, alkaliptosis, and oxeiptosis. It is plausible that additional genes regulating these types of PCD have been identified in recent studies. Future research should consider incorporating these newly discovered genes to enhance the comprehensiveness of the analysis. Additionally, only four GBM patients were included in the single-cell analysis, which may not be sufficient for the heterogeneity of GBM in the entire population. In conclusion, additional empirical studies are necessary to examine the fundamental biological roles and associations between risk forecasts and GBM. Although our model provides valuable insights, verifying these associations through experimental studies will contribute to a more comprehensive understanding of GBM biology with a larger population.

## Conclusion

In summary, our study demonstrated the applicability of PCD-related genes in classifying GBM patients based on diverse clinical and molecular characteristics. A new predictive model was created using 14 genes related to PCD, which demonstrated significant predictive capability by independently forecasting the risk of survival in both the derivation and validation groups. However, the underlying mechanisms connecting these prognostic genes to the biological functions of GBM are still poorly understood and require further investigation.

### Supplementary Information

Below is the link to the electronic supplementary material.

Supplementary file1 (XLSX 25 KB)

Supplementary file2 (TIF 4534 KB)

Supplementary file3 (TIF 2309 KB)

Supplementary file4 (TIF 2354 KB)

Supplementary file5 (TIF 3453 KB)

Supplementary file6 (TIF 1919 KB)

Supplementary file7 (TIF 4536 KB)

## Data Availability

The data used to support the findings of this study are available from the corresponding author upon request.
