# Supplementary figures and images for: Association Between Diverse Cell Death Patterns Related Gene Signature and Prognosis, Drug Sensitivity, and Immune Microenvironment in Glioblastoma
Source: J Mol Neurosci. 2024 Jan 12;74(1):10. doi: 10.1007/s12031-023-02181-4 (PMC10787010; doi:10.1007/s12031-023-02181-4)

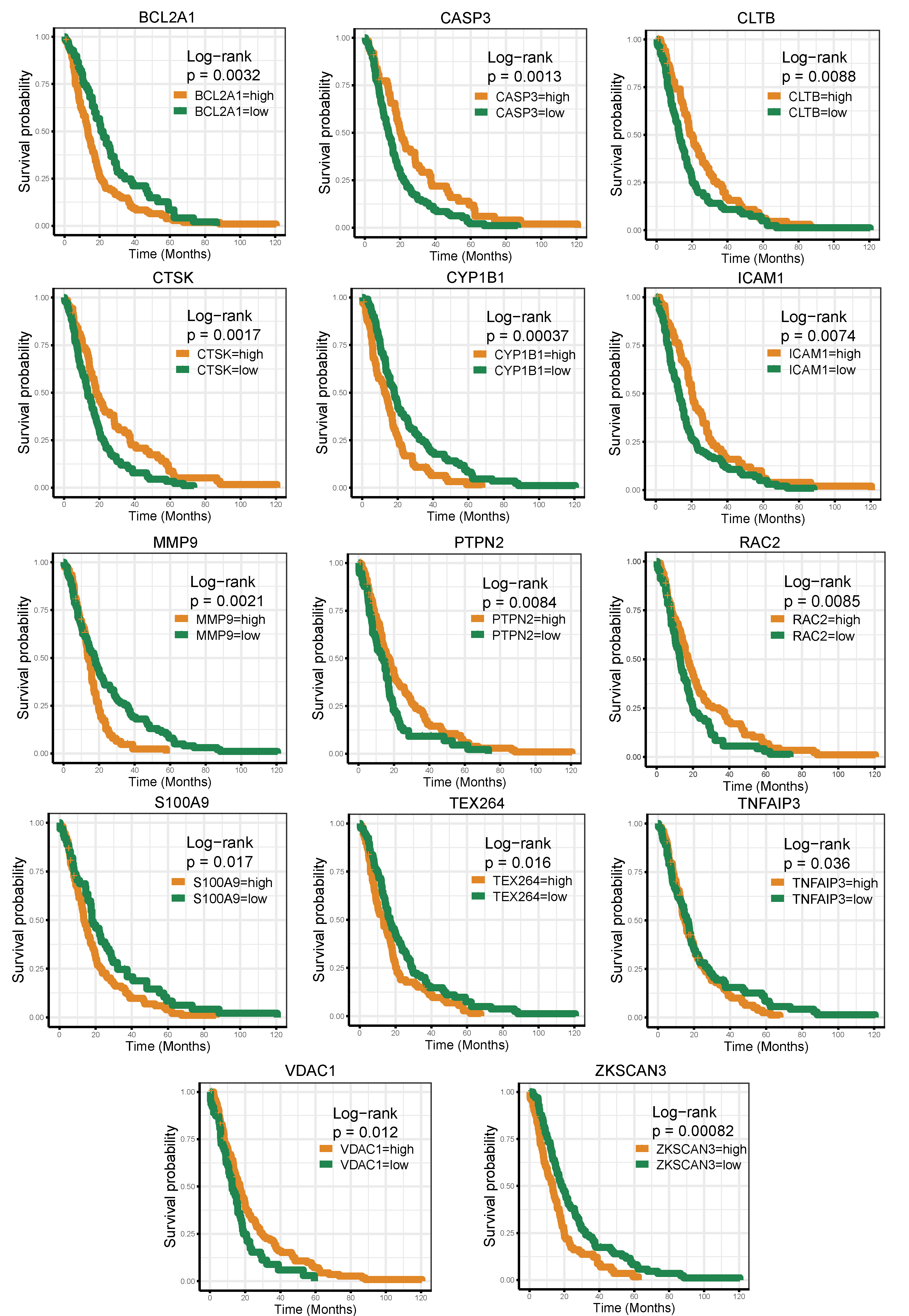

Supplement: Supplementary file 2 — Supplementary file2 (TIF 4534 KB) [file 12031_2023_2181_MOESM2_ESM.tif]

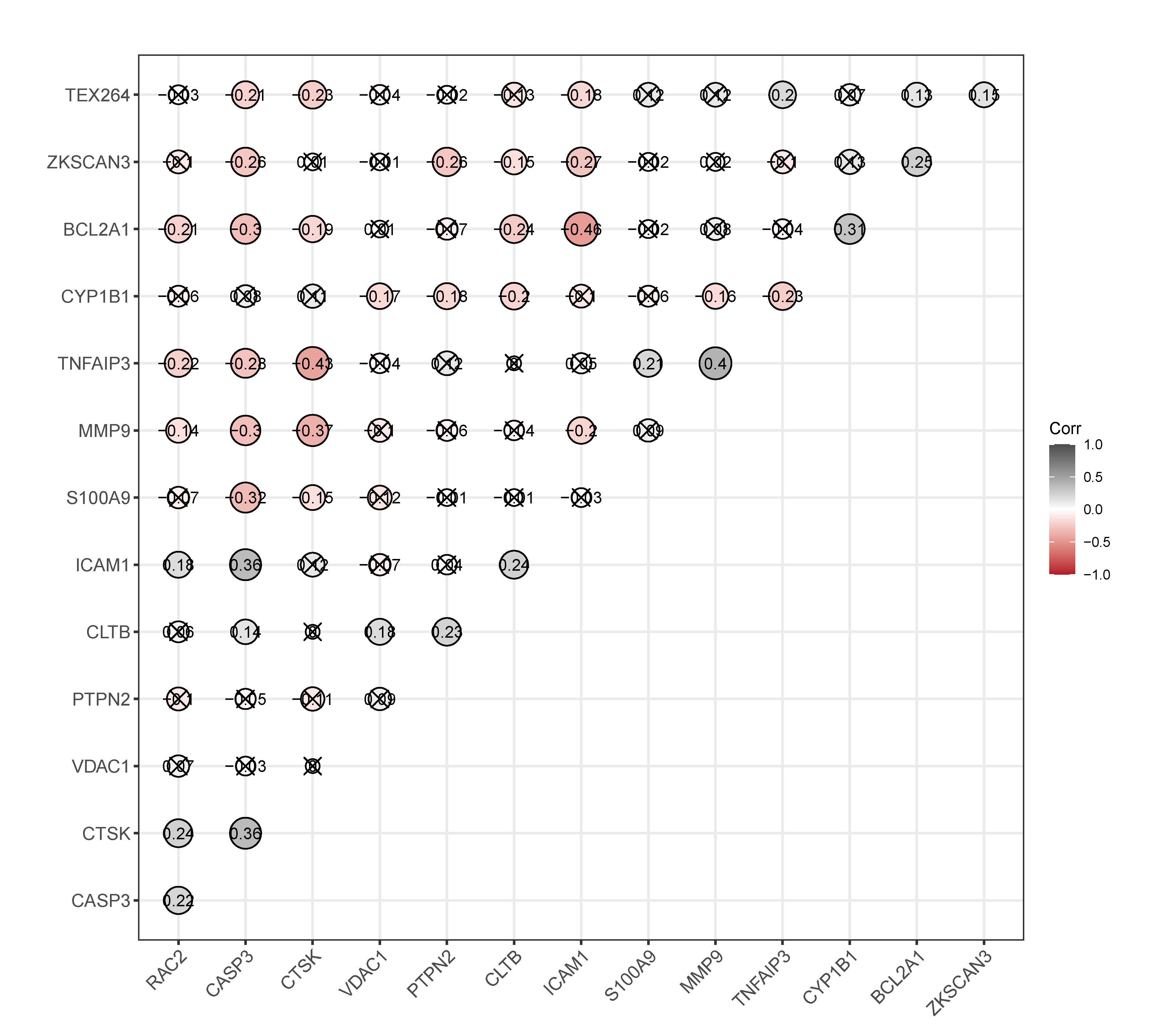

Supplement: Supplementary file 3 — Supplementary file3 (TIF 2309 KB) [file 12031_2023_2181_MOESM3_ESM.tif]

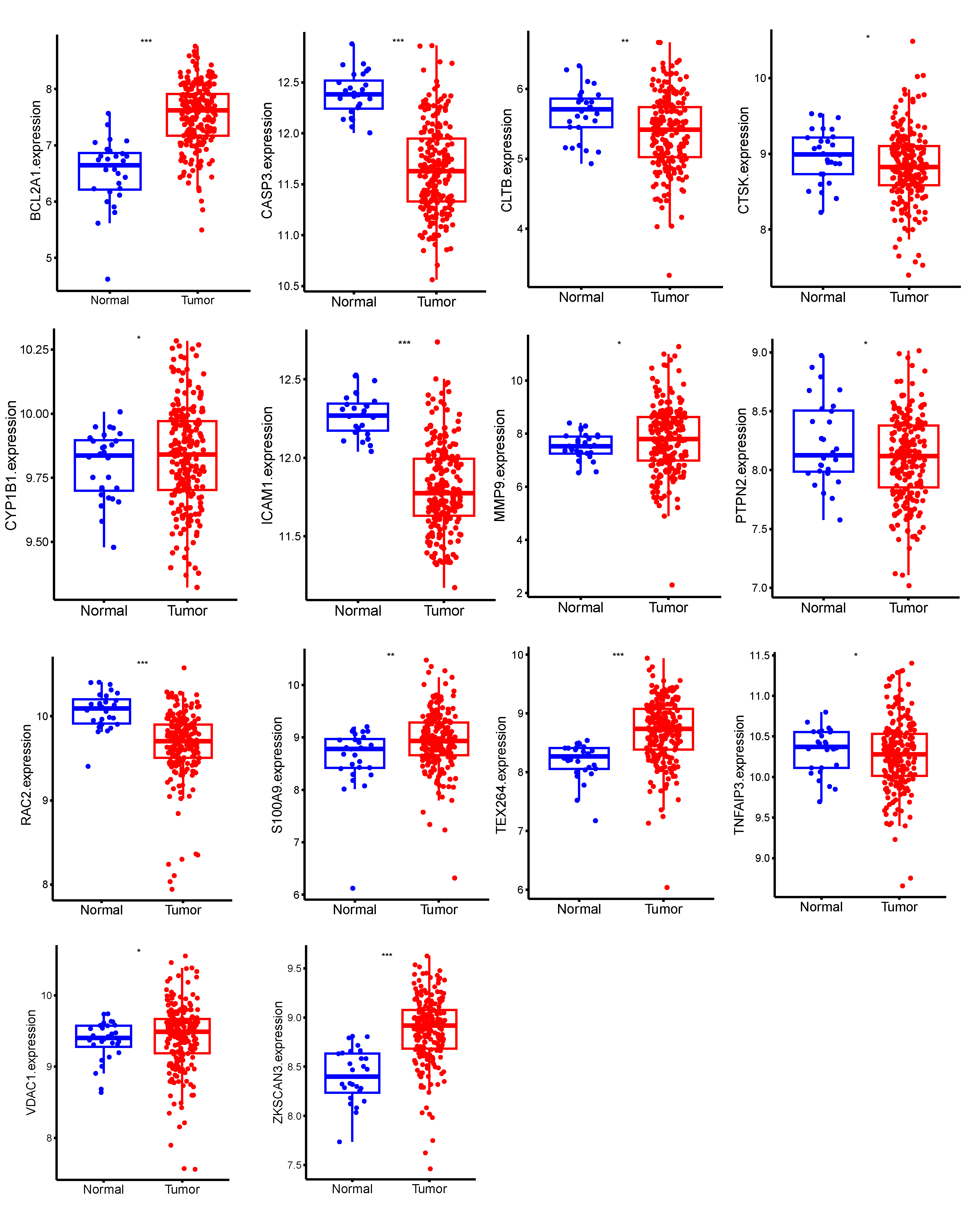

Supplement: Supplementary file 4 — Supplementary file4 (TIF 2354 KB) [file 12031_2023_2181_MOESM4_ESM.tif]

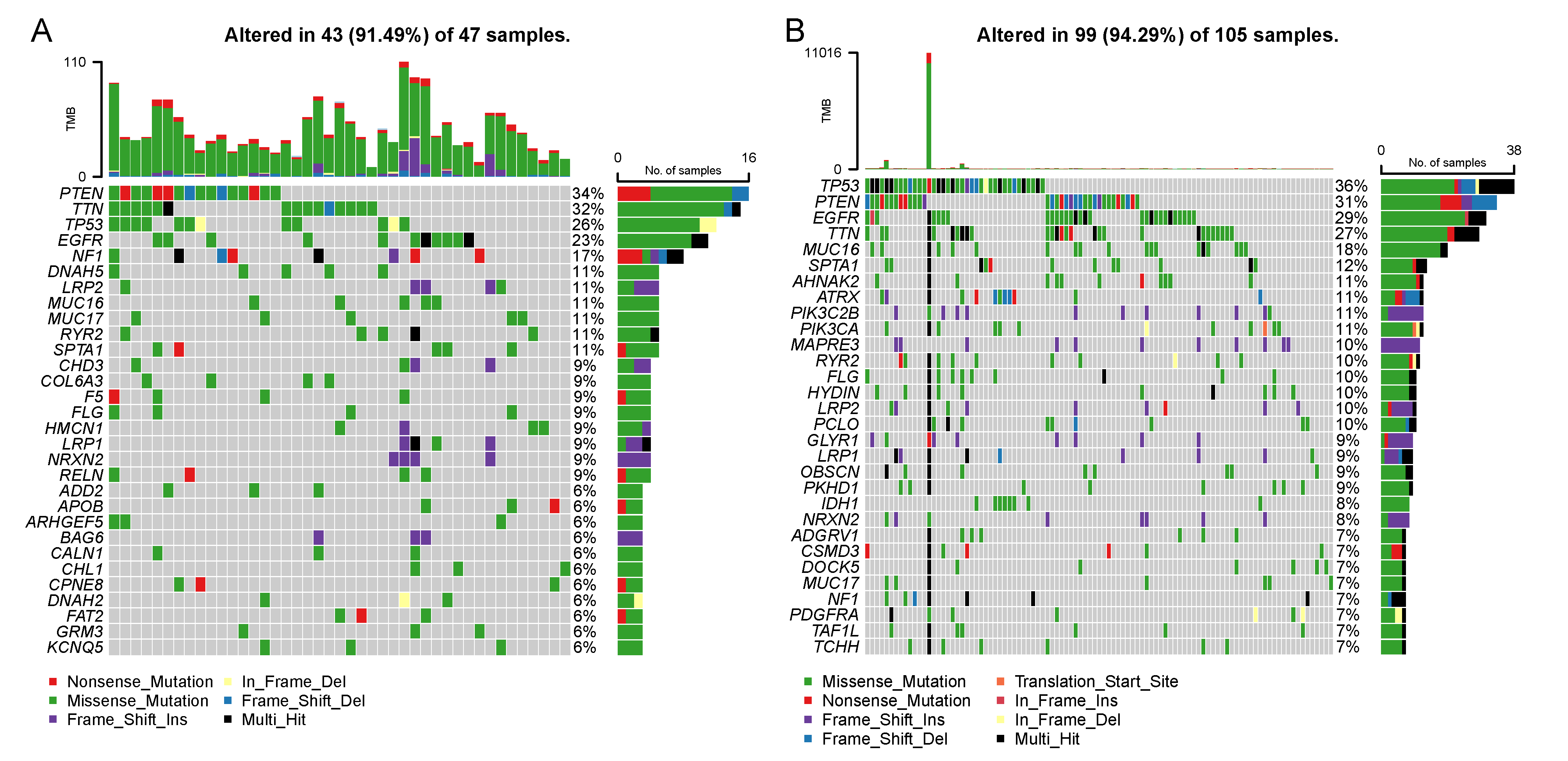

Supplement: Supplementary file 5 — Supplementary file5 (TIF 3453 KB) [file 12031_2023_2181_MOESM5_ESM.tif]

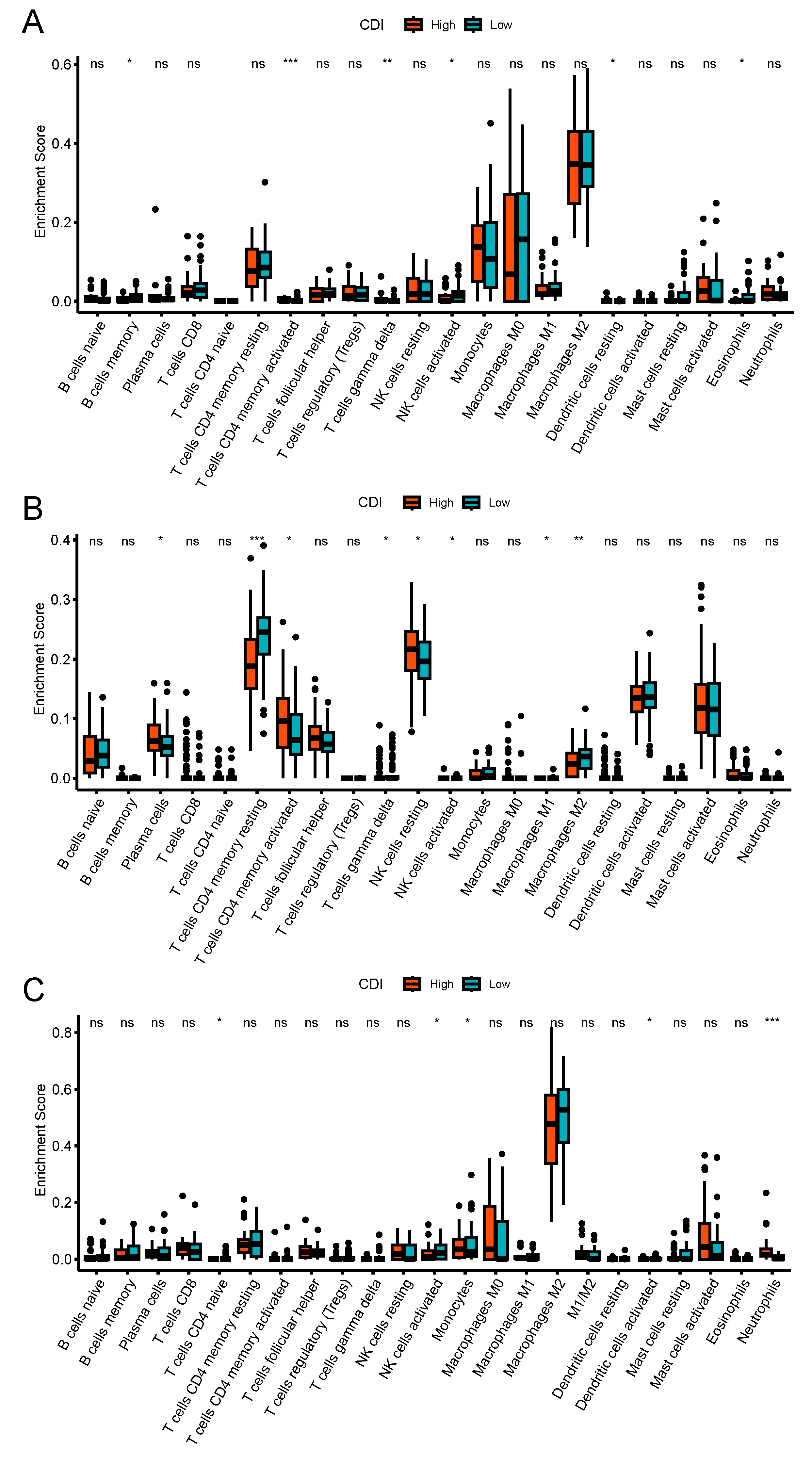

Supplement: Supplementary file 6 — Supplementary file6 (TIF 1919 KB) [file 12031_2023_2181_MOESM6_ESM.tif]

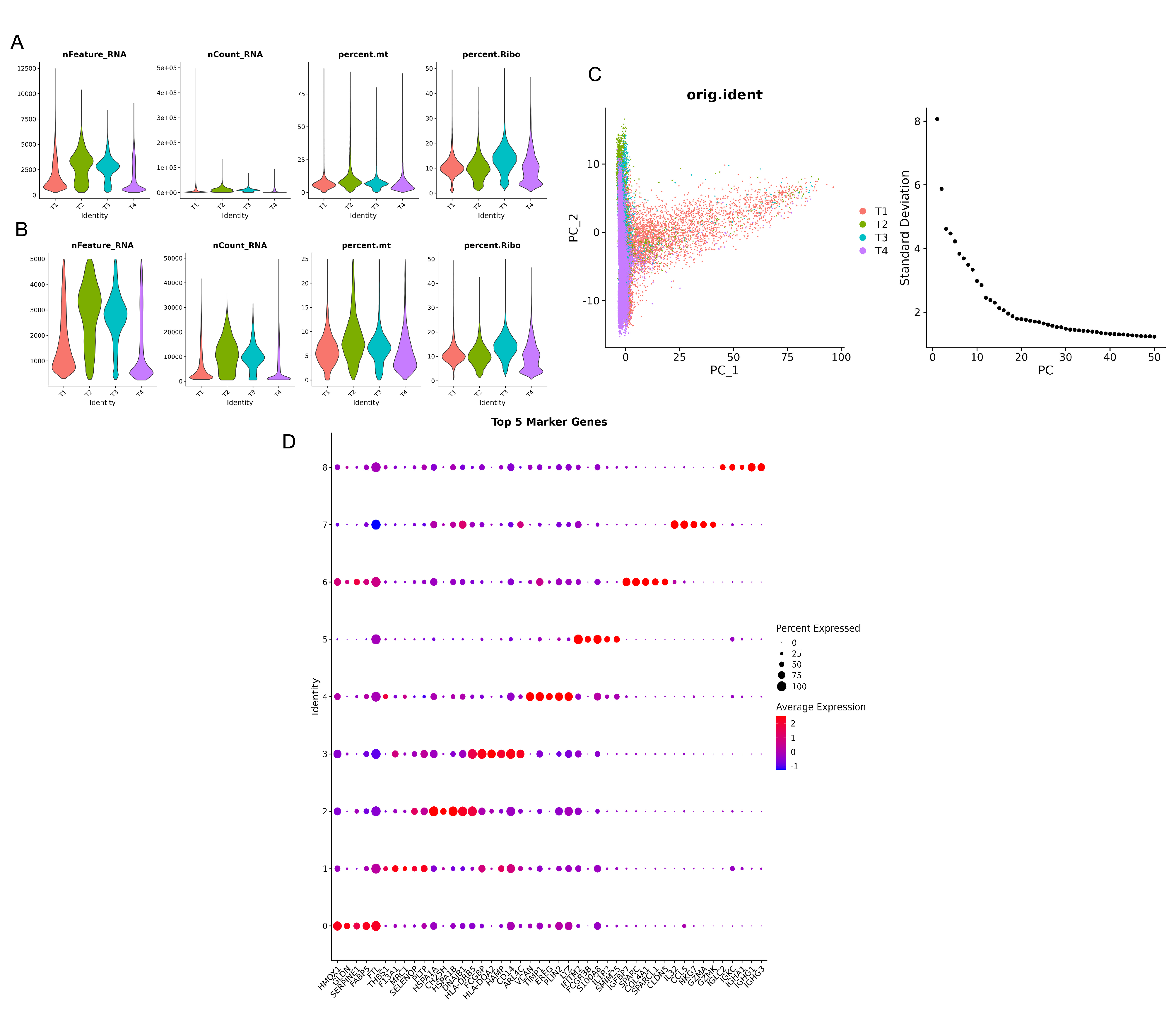

Supplement: Supplementary file 7 — Supplementary file7 (TIF 4536 KB) [file 12031_2023_2181_MOESM7_ESM.tif]
